# Overweight status is associated with extensive signs of microvascular dysfunction and cardiovascular risk

**DOI:** 10.1038/srep32282

**Published:** 2016-08-31

**Authors:** Sunni R. Patel, Srikanth Bellary, Said Karimzad, Doina Gherghel

**Affiliations:** 1 Vascular Research Laboratory, Ophthalmic Research Group, School of Life and Health Sciences, Aston University, Birmingham, UK; 2 Metabolic Medicine, Aston Research Centre for Healthy Ageing (ARCHA), Birmingham, UK

## Abstract

The aim of this present study was to investigate if overweight individuals exhibit signs of vascular dysfunction associated with a high risk for cardiovascular disease (CVD). One hundred lean and 100 overweight participants were recruited for the present study. Retinal microvascular function was assessed using the Dynamic Retinal Vessel Analyser (DVA), and systemic macrovascular function by means of flow-mediated dilation (FMD). Investigations also included body composition, carotid intimal-media thickness (c-IMT), ambulatory blood pressure monitoring (BP), fasting plasma glucose, triglycerides (TG), cholesterol levels (HDL-C and LDL-C), and plasma von Willebrand factor (vWF). Overweight individuals presented with higher right and left c-IMT (p = 0.005 and p = 0.002, respectively), average 24-h BP values (all p < 0.001), plasma glucose (p = 0.008), TG (p = 0.003), TG: HDL-C ratio (p = 0.010), and vWF levels (p = 0.004). Moreover, overweight individuals showed lower retinal arterial microvascular dilation (p = 0.039) and baseline-corrected flicker (bFR) responses (p = 0.022), as well as, prolonged dilation reaction time (RT, p = 0.047). These observations emphasise the importance of vascular screening and consideration of preventive interventions to decrease vascular risk in all individuals with adiposity above normal range.

Excess adipose tissue induces several metabolic changes, including dyslipidaemia, elevated blood pressure (BP), oxidative stress, and increased inflammation, thereby, contributing towards macro- and micro-vascular endothelial dysfunctions, increased arterial stiffness, and atherosclerosis^1^,^2^. These changes are traditionally associated with obesity and are not often reported in those with lower levels of adiposity that are classed as overweight. Indeed, it is usually presumed that the cardiovascular risk is not heightened in those with a body mass index (BMI) between 25–29.9 kg/m^2 ^3. Nevertheless, it is now thought that overweight individuals actually have an approximately 60% higher risk of cardiovascular disease (CVD) mortality when compared to age-matched lean controls^4^. Indeed, it has been demonstrated that even from early life, an increased BMI is associated, amonfg other changes, with low levels of nitric oxide (NO), the key contributor to normal vascular function^5^. Overweight children and young adults show evidence of macrovascular endothelial dysfunction and increased arterial stiffness^6^, which is reversible by increasing NO bioavailability and reduction of abdominal visceral fat^7^. It seems, therefore, that *any* excess adiposity would result in a higher chance of future cardiovascular risk.

It is well known that vascular endothelial dysfunction represents an early marker of atherosclerosis and precedes the clinical manifestations of CVD^8^. In addition, endothelial dysfunction affects the microcirculation much earlier than the macrocirculation in the course of vascular disease development^9^. This observation is extremely important. Due to the large total surface area of the microvessels, even an early activation of the endothelium at this level could have a large impact on bodily vascular health^10^. Consequently, detection of functional abnormalities at the microvascular level could serve as a better predictor for future risk of disease than testing macrovasculature.

Dynamic retinal vessel analysis (DVA) represents a non-invasive technique that assesses retinal microvascular motions in response to flickering light and by using this technique a diminished microvascular function has already been demonstrated in obese individuals^11^. However, no such studies have been carried out in overweight adults that are generally perceived as being at some risk for CVD but not at a level that would demand preventative care. Therefore, the aim of this study was to investigate micro- and macro-vascular function parameters and their relationship with established markers for future cardiovascular risk in otherwise healthy, overweight individuals compared to lean, age- and sex-matched controls.

## Methods

Written informed consent was obtained from all participants and ethical approval was granted from the local (Aston University) and NHS ethical committees (COREC West Midlands, UK). This study was designed and conducted in accordance with the tenets of the Declaration of Helsinki.

### Study Population

The study population consisted of healthy, normotensive White-European participants aged 30–55 years that were screened and recruited from the Health Clinics at Aston University, Birmingham, UK. Weight classifications were determined according to WHO definitions, whereby normal weight was classed as a BMI of 18.5–24.9 kg/m^2^ and overweight as a BMI of 25–29.9 kg/m^2^.

Subjects were excluded if they were classified as obese (BMI > 30 kg/m^2^), as well as, if they had a positive diagnosis of, or were taking medication for, cardio- or cerebro-vascular disease, coronary artery disease, heart failure, arrhythmia, stroke, transient ischaemic attacks, peripheral vascular disease, diabetes, hypertension or severe dyslipidaemia (defined as plasma TG > 6.00 mmol/L or cholesterol levels > 7.00 mmol/L). Smokers (including previous history of smoking) and all subjects taking vasoactive substances including dietary/vitamin/anti-oxidant supplementation, bronchodilators were also excluded from the present study. In addition, a standard 75 g oral glucose tolerance test (OGTT) was performed according to the WHO protocols on all participants a week prior to all other measurements and only normoglycaemic participants were asked to return for subsequent tests.

Furthermore, subjects were excluded if they had a refractive error of more than ±3 Dioptre Spherical (DS – depicting the convergent or divergent refractive power of the eye, i.e. hyperopia or myopia) and more than ±1 Dioptre Cylindrical (DC – depicts the degree of astigmatism, i.e. the curvature of the cornea and or lens of the eye) equivalent to minimise magnification error brought on by high refractive errors, and intraocular pressure (IOP) higher than 24 mmHg, cataract or any other media opacities, as well as, if they had a history of intraocular surgery or any form of retinal or neuro-ophthalmic disease affecting the ocular vascular system.

According to an already established procedure, when examining endothelial function, female participants were asked to fill in a validated menstrual cycle questionnaire and their investigations were carried out during the first week of the menstrual cycle (follicular phase)^12^.

### Investigations

All participants were screened for cardiovascular disease by a physician. Prior to the date of the study, participants were asked to fast and refrain from caffeine, alcohol, chocolate, and carbonated drinks, and to not exercise for 12 hours before the measurements were taken.

### General measurements

Anthropometric measures including height and weight were recorded using standard procedures. Body composition was measured using bioelectrical impedance (Biostat 220, Biospace, UK) to determine BMI, percentage body fat (PBF), waist-to-hip ratio (WHR), total fat mass, and fat free mass.

### Blood sampling and analyses

A qualified phlebotomist carried out the blood sampling, and all samples were obtained during the morning of the study, between 9:00 and 10:00 AM. Fasting plasma glucose, TG, total and HDL-C were measured using standard routine laboratory techniques using the Reflotron Desktop Analyser (Roche Diagnostics, UK). The TG/HDL cholesterol ratio^13^ and Total/HDL cholesterol ratio alongside Framingham score as a means of cardiovascular risk were also determined from the above values^14^.

Laboratory-validated protocols^15^ for in-house ELISA-testing were adopted to carry out plasma sampling of von-Willebrand Factor (vWF) to investigate possible signs of endothelial damage in overweight individuals.

### Micro- and macro-vascular studies

#### Ambulatory Blood Pressure

Ambulatory BP was measured using a 24-hour computer-operated ambulatory BP and electrocardiography (ECG) monitor (Cardiotens-01, Meditech Ltd., Hungary) for each subject. Measurements were performed in ambulatory conditions and programmed to measure BP oscillometrically every 15 minutes during the subject’s active period and every 30 minutes during the passive period. The 24-hour data was later downloaded and systolic BP (SBP), diastolic BP (DBP) and mean BP [MBP ≈ (⅔ X DBP) + (⅓ X SBP)], were calculated using the Medibase software (Meditech Version 1.42).

#### Intima-media thickness

Real-time intima-media thickness measurements for both carotid arteries were obtained for all participants through analysis of ultrasound images taken from the right and left common carotid arteries at the neck using a high-resolution B-mode ultrasound system (Acuson Sequoia, 5 MHz linear transducer, Siemens, USA) according to an already published protocol^16^.

#### Retinal vascular function

Retinal vessel reactivity was measured with the dynamic retinal vessel analyser (DVA, IMEDOS GmbH, Jena, Germany) using an already established and recommended protocol^17^. All measurements were performed in one randomly selected eye for each subject between 8:00 and 11:00 AM, and in a quiet, temperature-controlled room (22 °C)^17^,^18^. Retinal vessel reactivity, in the form of arterial and venous reaction time (RT) and maximum diameter (MD), were determined using our newly defined method of Sequential and Diameter Response Analysis (SDRA), which is described elsewhere^19^.

**Figure 1:**
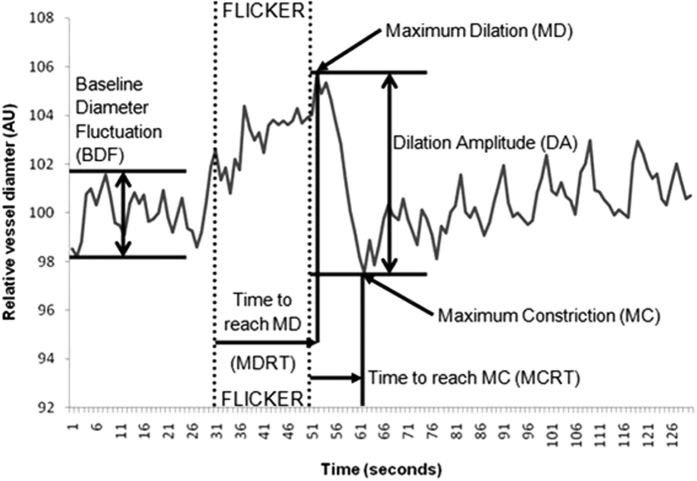
The retinal vessel reactivity and time course components used in SDRA Figure adapted with consent from author. The copyright of the original image rests with the Association for Research in Vision and Ophthalmology^19^.

The following retinal vessel reactivity and time course parameters (using one-second averaged data for 3 flicker cycles) were calculated (Fig. 1): baseline-diameter fluctuation (BDF), as the difference between maximum and minimum baseline vessel diameter; the maximum diameter (MD), as the maximal vessel dilation in response to flicker light stimulation expressed as a percentage from baseline; the MD reaction time (MDRT), as the time taken (seconds) to reach the maximum vessel diameter during the 20-second flicker exposure; the maximum constriction (MC), as the percentage to baseline minimal vessel diameter within 30 seconds of the recovery period; and the maximum constriction reaction time (MCRT), as the time taken (seconds) to reach maximal vessel constriction. In addition, the dilation amplitude (DA), as the difference between maximal dilation and constriction responses, and the baseline-corrected flicker response (bFR), which accounted for the BDF were also determined^19^.

#### Brachial artery Flow Mediated Dilation (FMD)

Brachial artery flow-mediated dilation (FMD) was measured using high-resolution ultrasonography, with a 7 mm 8 MHz linear-array (Siemens; Acuson Sequoia, UK). Briefly, vessel diameter was continuously measured, from the anterior to the posterior interface between the media and adventitia, using specialised wall-detection and artificial neural networking software (VIA^®^ Software, UK), and the data was recorded on a personal computer. The procedure was carried out according to a previously validated and universally accepted protocol comprising of a 2-minute baseline recording, followed by 5 minutes of occlusion, and a 2-minute recovery period. Following a subsequent 10-minute re-acclimatisation period, an exogenous NO donor (300 mcg sublingual glyceryl trinitrate – GTN tablet) was administered. The FMD% was determined as absolute change from baseline using real-time raw data, as well as, GTN-induced changes^20^.

Numerous factors have been known to contribute to the variability of FMD, mainly equipment- and operator-related, as well as, physiological influences. To minimise equipment- and operator-related influences, a standardised protocol was adhered to, and all analyses were performed off-line by a blinded observer. To minimise physiological influences known to exert profound effects on endothelial function, i.e., diurnal variation and dietary substances, we further standardised environmental factors (temperature, noise, and excluded the use of vasoactive substances.

### Statistical analysis

All analyses were performed using Statistica^®^ software (StatSoft Inc.; Version 9, USA). Prior to any analysis, all data was tested for normal distribution and thus a suitable test was adopted. Differences in mean values for each of the measured biochemical, demographic, and anthropometric variables were compared by independent samples t-test for continuous variables. A multivariate analysis was performed to test the influence of BMI, BP, and circulating markers on the measured variables. Comparison of retinal vessel reactivity for each of the 3 repeated flicker periods was made by Friedman analysis of variance (ANOVA) following within-group analysis as the data was not normally distributed even following log transformation. Differences in brachial artery and FMD responses (also not normally distributed) were assessed by Kruskal-Wallis testing. Differences between groups in retinal and systemic vascular function were computed by analysis of variance (ANOVA) or covariance (ANCOVA) where appropriate. A p value of <0.05 was considered statistically significant for the retinal and brachial parameters, and a stricter criteria was adopted for within-group and multiple comparisons of the biochemical and demographic variables (p ≤ 0.01 to account for multiple comparisons and thereby minimise bias towards Type II errors).

## Results

In the present study, an initial 226 individuals were screened for inclusion, following which 26 were excluded as they were diagnosed with impaired glucose tolerance and referred back to appropriate outpatient management. A final 200 subjects were recruited, based on the diagnosis criteria, and allocated to either the normal (n = 100; men = 48, women = 52) or overweight (n = 100; men = 51, women = 49) group. As evident in Table 1, there were no statistically significant differences between the two groups with regards to age and gender distribution of the participants (p > 0.05).

The anthropometric characteristics of the normal and overweight group are also presented in Table 1. Compared to lean individuals, overweight subjects showed significantly greater right and left c-IMT (p = 0.005 and p = 0.002, respectively), 24-hr BP values (SBP, DBP, MBP, all p < 0.001), fasting glucose (p = 0.008) and TG (p = 0.003) levels, alongside higher TG: HDL-C ratio (p = 0.010). In addition, overweight individuals exhibited higher body composition indices (weight, BMI, WHR, PBF, fat mass, all p < 0.001) and plasma levels of vWF (p = 0.01) than the age-matched controls.

### Microvascular function

A multiple regression analysis found that weight and BMI had a positive effect on retinal arterial MD (b = 0.203; p = 0.017, and b = 0.190; p = 0.025, respectively), but not on the other measured microvascular parameters.

The measured retinal parameters presented in Table 2 indicate that, after correcting for all influential variables (BMI, BP), overweight individuals exhibited a reduced arterial MD (p = 0.039) and bFR (p = 0.022), as well as, an increased MDRT (p = 0.047) compared to the lean subjects. There were no differences in retinal venous reaction parameters between the two study groups (all p > 0.05).

### Macrovascular function

The brachial artery results presented in Table 3 show that after correcting for influential variables (BMI, BP), the baseline vessel diameter was larger in the overweight individuals when compared to the age- and gender matched lean controls (p = 0.018). All the other measured brachial parameters were, however, comparable between the two groups (p > 0.05).

## Discussion

This study demonstrates, for the first time, that otherwise healthy overweight individuals present with signs of microvascular functional impairment, as well as, increased circulatory plasma markers of endothelial dysfunction when compared to lean individuals. Although presenting with a larger baseline diameter, the brachial artery function was not affected in our overweight individuals cohort.

Consistent with previous research^21^, the present study demonstrates that overweight subjects show elevated BP values, higher TG, cholesterol, and fasting blood glucose. All these factors either separate or in conjunction, are well known contributors to cardiovascular risk and pre-diabetes in both adults and children^7^,^21^. In particular, triglyceride-rich lipoprotein remnants may cause endothelial damage^22^. In addition, the TG: HDL-C ratio was also increased in our overweight subjects and this parameter is a known independent determinant of increased arterial stiffness^23^. An elevated TG: HDL-C ratio has also been shown to have a strong correlation with fasting plasma insulin concentrations and when used alongside TG levels, represents a good surrogate marker for the presence of insulin resistance (IR)^24^. It is known that abnormal IR plays an important role in the development of endothelial dysfunction through a reduced production of NO, as well as, by accelerating the onset of atherosclerosis^25^. Indeed, our overweight cohort demonstrated signs of pre-clinical atherosclerotic changes as measured by c-IMT and a modified baseline diameter of the brachiar artery, as measured by FMD; some of these parameters are well-known predictors for future cardiovascular complications^26^,^27^,^28^. However, we have not observed any correlation between c-IMT and FMD changes and this could further support previous assumptions that in younger patients without overt disease, but with certain risk for cardiovascular pathologies, there is no direct relationship between functional (FMD) and structural(c-ITM) vascular modifications^29^. Indeed, vascular dysfunction is known to precede the occurrence of atherosclerosis and signals the risk for future vascular disease^30^. Our overweight group also demonstrated abnormal vWF levels, possibly showing pathophysiological changes of the vascular endothelium. Nevertheless, neither of the measured macrovascular parameters correlated with this circulatory parameter.

An important finding of the present study was the detection of an impaired retinal vessel function in response to flickering stimulus (a reduced bFR and arterial MD and an increased MDRT) in overweight but not in lean individuals. It has been previously reported that a high BMI can be associated with narrower retinal arteriolar calibres^31^,^32^. In addition, complex alterations of the retinal microvascular function were already observed in obese individuals of various age groups^11^,^33^. Nevertheless, to our knowledge, this is the first report showing functional retinal vascular changes *in overweight but not yet obese individuals*, therefore, opening a new opportunity for early detection and prevention of cardiovascular complication in this type of population. Although the precise mechanisms behind our results need further elucidation we can, however, formulate some hypotheses. For instance, there is evidence to suggest that the changes observed in our measured MD and MDRT parameters could be the result of either early atherosclerosis, increased arterial stiffness, or reduced NO bioavailability to peripheral tissues^34^. Indeed, a high BMI has been found to be associated with reduced NO^5^ and increased levels of vasoconstrictor substances such as endothelin-1 (ET-1) and angiotensin-II (Ang-II)^35^. Therefore, our observation showing that both weight and BMI had significant influences on retinal arterial MD parameters could possibly be explained by the detrimental effect of BMI on the vascular dilatory capacity of the retinal microvessels through the above mentioned mechanisms. Although neither NO or vasoconstrictor substances were measured in the present study, one could hypothesize that abnormal levels of vasodilatory or vasoconstrictory mediators are, at least in part, responsible for our findings. More research is necessary to confirm our hypothesis.

Despite recording a modified baseline diameter of the brachial artery, we could not demonstrate any signs of macrovascular dysfunction in our overweight cohort. The literature reports conflicting results on the effect of overweight abd obesity on macrovascular function^6^,^36^,^37^,^38^. It is possible that the vascular effect of the adipokines secrated by the perivascular adipose tissue (PVAT) at this level varies according to the degree of obesity^39^. Moreover, in physiological conditions, the PVAT can also have a vasorelaxant effect with a protective role^40^. This effect could have been responsible for our finding of an increased brachial artery baseline diameter in our overweight individuals it which PVAT was not yet dysfunctional enough to exert detrimental effects on the macrovascular function^41^. Nevertheless, the macro- and microvascular beds’ functions are governed by different physiological mechanisms. Moreover, it is the microcirculation that may be a better target for the assessment of risk as it is the microvessels that are considered to be the first to be affected in the course of vascular disease^9^. Therefore, our finding of abnormal vascular function at the retinal microvascular level in overweight individuals without any overt diseases is relevant and points towards the need for a much earlier screening for vascular detrimental effects that any excess adiposity could have.

As discussed above, microvascular dysfunction can be trigerred in areas with PVAT by subclinical inflammation mediated via adipokines/cytokines and infiltrating macropahges^42^. Nevertheless, these molecules can also trigger remote inflammatory effects withy potential vascular changes as far as the retinal circulation, usually devoided of PVAT, therefore, resulting in abnormal vascular function at this level^43^. Further research is necessary to elucidate this point.

Dietary and social factors (such as ingestion of high saturated fats or lack of exercise) have not been assessed in the present study and this could therefore be perceived as a limitation. Moreover, larger studies are needed to investigate the effect of other possible confounding factors such as insulin sensitivity and menopause status. Nevertheless, even in the presence of these limitations, the results of our study show that in overweight but not yet obese individuals, signs of microvascular dysfunction exist alongside with abnormal circulatory levels of cardiovascular risk markers. We suggest that the assessment of retinal microcirculation may be a good, easy and non-invasive alternative for assessing vascular dysfunction associated with excess adiposity^44^. Therefore, the clinical relevance of screening for early CVD risk in overweight individuals using this method is supported by these preliminary, but promising results.

## Additional Information

**How to cite this article**: Patel, S. R. *et al.* Overweight status is associated with extensive signs of microvascular dysfunction and cardiovascular risk. *Sci. Rep.* **6**, 32282; doi: 10.1038/srep32282 (2016).

## Figures and Tables

**Table 1:** Baseline data for lean and overweight groups.

	Mean (SD)		p-value
	Control group[n = 50]	Overweight Group[n = 50]	
Demographic Data			
Age (years)	41.7 (11.1)	42.2 (10.6)	
SBP (mmHg)	114 (14)	123 (12)*	<0.001
DBP (mmHg)	72 (10)	81 (9)*	0.001
MAP (mmHg)	86 (10)	92 (9)*	<0.001
IOP (mmHg)	13 (3)	14 ± (2)	0.411
Bodycomposition Data			
Weight (kg)	65.4 (9.4)	78.5 (9.3)*	**<0.001**
BMI (kg/m^2^)	22.4 (3.1)	27.4 (1.3)*	**<0.001**
WHR (AU)	0.90 (0.14)	0.95 (0.03)*	**<0.001**
PBF (%)	25.8 (7.1)	32.1 (7.6)*	**<0.001**
Fat Mass (kg)	16.8 (5.3)	24.8 (4.7)*	**<0.001**
Fat Free Mass (kg)	48.7 (9.2)	53.7 (10.7)*	**<0.001**
Metabolic Data			
Glucose (mmol/L)	4.78 (0.75)	5.12 (0.64)*	**0.008**
2 hour GTT (mmol/L)	6.67 (2.29)	7.33 (2.14)	0.146
TG (mmol/L)	1.08 (0.41)	1.42 (0.79)*	**0.003**
HDL Cholesterol (mmol/L)	1.27 (0.41)	1.14 (0.38)	0.065
LDL Cholesterol (mmol/L)	2.53 (0.79)	2.74 (0.88)	0.142
Total Cholesterol (mmol/L)	4.30 (0.83)	4.53 (0.97)	0.142
TG: HDL-C (mmol/L)	2.36 (2.06)	3.37 (2.95)*	**0.01**
CVD Risk Data			
R-IMT (mm)	0.052 (0.015)	0.058 (0.014)	**0.005**
L-IMT (mm)	0.053 (0.016)	0.064 (0.015)*	**0.002**
Framingham Score (%)	1.7 (2.4)	2.1 (3.4)	**0.479**
Total: HDL-C (mmol/L)	3.70 (1.32)	4.36 (1.57)*	**0.01**
Biochemical Data			
vWF (μ/dL)	112.37 (58.67)	141.24 (55.36)*	**0.01**

Values quoted in mean ± SD. SBP: systolic blood pressure; DBP: diastolic blood pressure; MAP: mean arterial pressure; IOP: intraocular pressure; BMI: body mass index; WHR: waist-to-hip ratio; PBF: percentage body fat; GTT: glucose tolerance test; TG: triglyceride; HDL: high-density lipoprotein; LDL: low-density lipoprotein; TG: HDL-C: TG-to-HDL ratio; CVD: cardiovascular disease; R-IMT: right intima media thickness L-IMT: left intima media thickness; vWF: von Willebrand factor. *Significant differences when compared to normal weight controls (p < 0.01).

**Table 2:** Retinal arterial and venous measures for both groups.

	Mean (SD)		p-value
	Control group[n = 50]	Overweight Group[n = 50]	
Artery			
AD (AU)	121.59 (112.40–132.60)	120.96 (109.19–131.22)	0.09
BDF (AU)	5.71 (3.75–7.09)	5.04 (2.77–6.67)	0.129
MD (%)	5.55 (3.66–7.12)	**4.54** (2.80–5.98)*	**0.039**
MDRT (secs)	16.9 (12.3–20.7)	**20.5** (14.0–24.0)*	**0.047**
MC (%)	3.24 (1.66–4.57)	3.14(1.58–4.59)	0.526
MCRT (secs)	19.7 (17.3–23.3)	20.3 (17.2–23.2)	0.222
DA (AU)	8.78 (6.78–10.18)	7.68 (5.24–9.00)	0.053
bFR (%)	3.76 (1.02–4.83)	**2.12** (0.43–4.05)*	0.022
Vein			
AD (AU)	156.73 (139.18–173.78)	156.00 (142.47–166.35)	0.471
BDF (AU)	4.13 (2.56–5.12)	4.29 (2.68–5.31)	0.461
MD (%)	5.59 (4.44–6.54)	5.64 (3.87–6.71)	0.433
MDRT (secs)	20.1 (17.3–22.7)	19.9 (17.0–23.2)	0.789
MC (%)	1.44 (0.33–4.84)	1.75 (0.63–2.16)	0.665
MCRT (secs)	21.8 (19.7–25.3)	21.1 (18.6–23.7)	0.525
DA (AU)	7.27 (5.33–9.00)	7.39 (4.83–9.31)	0.323
bFR (%)	3.12 (1.74–4.40)	3.16 (1.13–4.91)	0.425

Average values corrected for CVD risk markers quoted in mean (IQR). AU: arbitrary units; AD: absolute diameter; BDF: baseline diameter fluctuation; MD: maximum dilation; MDRT: reaction time to reach maximum diameter to flicker stimulation; MC: maximum constriction; MCRT: reaction time to maximum constriction post flicker; DA: dilation amplitude; bFR: baseline-corrected flicker response. *Significant values in bold (p < 0.05, ANCOVA).

**Table 3:** FMD data between lean and overweight individuals.

	Mean (SD)		p-value
	Control group[n = 50]	Overweight Group[n = 50]	
BRACHIAL ARTERY			
Baseline Diameter (mm)	4.00 (3.20–4.31)	**4.39** (3.75–4.77)*	**0.018**
Peak Diameter (mm)	4.40 (3.54–4.69)	4.68 (4.04–5.18)	0.163
FMD (%)	7.10 (3.66–11.94)	5.15 (2.67–9.47)	0.134
GTN			
GTN-Peak (mm)	4.99 (4.13–5.64)	5.52 (4.75–5.61)	0.054
GID (%)	23.07 (18.59–30.99)	23.96 (14.16–27.25)	0.829
FMD/GID (%)	0.23 (0.05–0.44)	0.17 (0.12–0.55)	0.823

Average values quoted in mean (IQR). AD: absolute diameter; MD: maximum diameter; FMD: flow-mediation dilation response; GID: GTN-induced dilation; FMD/GID: FMD/GID ratio. *Significant values in bold (p < 0.05).
